# Supplementary material for: Generation and Purification of RANKL-Derived Small-Fragment Variants for Osteoclast Inhibition
Source: Pharmaceutics. 2025 Oct 25;17(11):1385. doi: 10.3390/pharmaceutics17111385 (PMC12655559; doi:10.3390/pharmaceutics17111385)
Supplement: Supplementary file 1 [file pharmaceutics-17-01385-s001.zip › pharmaceutics-3816085-supplementary.pdf]

Colorimetric cell viability assay

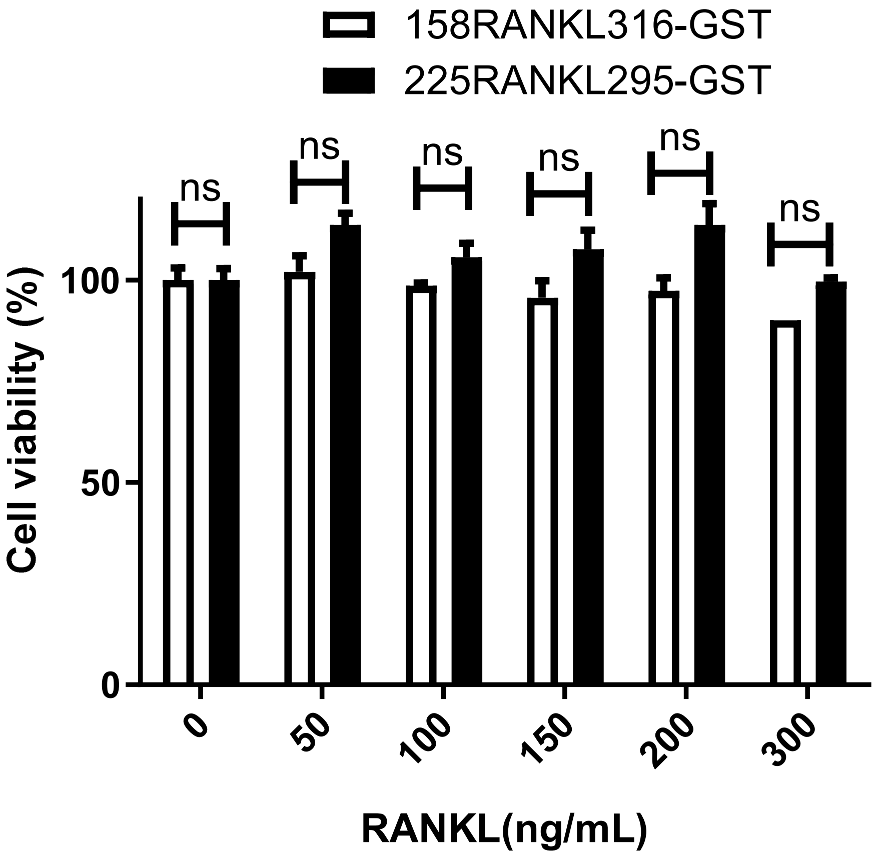

Figure S1. The cell viability in presence with 158RANKL316-GST or 225RANKL295-GST in Bone marrow–derived macrophages (BMM; 1 × 10^4 cells/well) cells. The cells were seeded into 96-well plates. Cells were co-treated with M-CSF (60 ng/mL) and either 158RANKL316-GST or 225RANKL295-GST at 0, 50, 100, 150, 200, or 300 ng/mL, and incubated for 1 days. After incubation, 110 µL of EZ-Cytox reagent (DAEILLAB Co. Seoul, Korea) was added to each well , according to manufacture's protocol and allowed to react for 1 hr at 37 °C, followed by measurement of absorbance at 450 nm using a microplate reader. Ns; Not significant.
